# Targeted hematopoietic stem cell depletion through SCF-blockade

**DOI:** 10.1186/s13287-024-03981-0

**Published:** 2024-10-29

**Authors:** Yan Yi Chan, Pui Yan Ho, Carla Dib, Leah Swartzrock, Maire Rayburn, Hana Willner, Ethan Ko, Katie Ho, Julian D. Down, Adam C. Wilkinson, Hiro Nakauchi, Morgane Denis, Taylor Cool, Agnieszka Czechowicz

**Affiliations:** 1 Division of Hematology, Oncology, Stem Cell Transplantation and Regenerative Medicine, Department of Pediatrics, Stanford University School of Medicine, Stanford, CA 94305 USA; 2 Institute for Stem Cell Biology and Regenerative Medicine, Stanford University School of Medicine, Stanford, CA 94305 USA; 3 Center for Definitive and Curative Medicine, Stanford University School of Medicine, Stanford, CA 94305 USA; 4 Koch Institute for Integrative Cancer Research, Massachusetts Institute of Technology, Cambridge, MA 02139 USA; 5 Radcliffe Department of Medicine, MRC Weatherall Institute of Molecular Medicine, University of Oxford, Oxford, UK

## Abstract

**Background:**

Hematopoietic stem cell transplantation (HSCT) is a curative treatment for many diverse blood and immune diseases. However, HSCT regimens currently commonly utilize genotoxic chemotherapy and/or total body irradiation (TBI) conditioning which causes significant morbidity and mortality through inducing broad tissue damage triggering infections, graft vs. host disease, infertility, and secondary cancers. We previously demonstrated that targeted monoclonal antibody (mAb)-based HSC depletion with anti(α)-CD117 mAbs could be an effective alternative conditioning approach for HSCT without toxicity in severe combined immunodeficiency (SCID) mouse models, which has prompted parallel clinical αCD117 mAbs to be developed and tested as conditioning agents in clinical trials starting with treatment of patients with SCID. Subsequent efforts have built upon this work to develop various combination approaches, though none are optimal and how any of these mAbs fully function is unknown.

**Methods:**

To improve efficacy of mAb-based conditioning as a stand-alone conditioning approach for all HSCT settings, it is critical to understand the mechanistic action of αCD117 mAbs on HSCs. Here, we compare the antagonistic properties of αCD117 mAb clones including ACK2, 2B8, and 3C11 as well as ACK2 fragments in vitro and in vivo in both SCID and wildtype (WT) mouse models. Further, to augment efficacy, combination regimens were also explored.

**Results:**

We confirm that only ACK2 inhibits SCF binding fully and prevents HSC proliferation in vitro. Further, we verify that this corresponds to HSC depletion in vivo and donor engraftment post HSCT in SCID mice. We also show that SCF-blocking αCD117 mAb fragment derivatives retain similar HSC depletion capacity with enhanced engraftment post HSCT in SCID settings, but only full αCD117 mAb ACK2 in combination with αCD47 mAb enables enhanced donor HSC engraftment in WT settings, highlighting that the Fc region is not required for single-agent efficacy in SCID settings but is required in immunocompetent settings. This combination was the only non-genotoxic conditioning approach that enabled robust donor engraftment post HSCT in WT mice.

**Conclusion:**

These findings shed new insights into the mechanism of αCD117 mAb-mediated HSC depletion. Further, they highlight multiple approaches for efficacy in SCID settings and optimal combinations for WT settings. This work is likely to aid in the development of clinical non-genotoxic HSCT conditioning approaches that could benefit millions of people world-wide.

**Supplementary Information:**

The online version contains supplementary material available at 10.1186/s13287-024-03981-0.

## Introduction

Given the unique ability of hematopoietic stem cells (HSCs) to self-renewal for life and replenish the entire hematolymphoid system, hematopoietic stem cell transplantation (HSCT) has become a widely used treatment for patients with many different genetic, malignant, and autoimmune blood immune diseases using both allogeneic and autologous grafts [1]. However, despite the broad curative potential of HSCT that has been further increased through the advent of HSC-based gene therapy, use of HSCT today is still primarily limited to patients with cancer or other terminal diseases because of the toxic conditioning required to enable HSCT. Currently, the standard of care for HSCT requires high-doses of genotoxic chemotherapy and/or irradiation conditioning before HSCT which induces non-specific tissue damage resulting in high morbidity and mortality [2]. In particular, these genotoxic agents directly cause debilitating side effects in patients including infections, mucositis, multi-organ damage, infertility, and secondary malignancies [2–4]. Furthermore, mounting evidence also supports that tissue inflammation from genotoxic conditioning primes patients to develop graft versus host disease (GvHD) after allogeneic HSCT [5]. Replacing genotoxic conditioning with safe agents that have equivalent efficacy could dramatically expand HSCT use and benefit millions of patients that are affected by diverse blood and immune diseases.

Anti(α)-CD117 monoclonal antibody (mAb)-mediated conditioning has been developed as a safer alternative to genotoxic conditioning for allogeneic or autologous gene-modified HSCT. CD117, also known as c-kit, is a receptor for the cytokine stem cell factor (SCF) which is an essential factor for HSC growth and proliferation [6–8]. A variety of αCD117 mAbs have been developed to CD117 by many different groups over the years [9, 10]. However, we have previously shown in immunodeficient mice modeling severe combined immunodeficiency disease (SCID) that α-mouse(m) CD117 mAb ACK2 uniquely enables host HSC depletion and enhances donor engraftment as a stand-alone conditioning agent pre-HSCT without need for additional genotoxic chemotherapy or irradiation treatment [11]. This work has been the foundation for the development of clinical-grade human αCD117 mAb AMG191, now named briquilimab, as a clinical conditioning agent for patients [12, 13]. Initial safety and efficacy results with this agent have been reported in a clinical trial in SCID patients (NCT02963064) [14, 15], however efficacy has varied between patients and has only been reported in T-, B- immunodeficient patients. Subsequent clinical trials using briquilimab conditioning for other disease indications ranging from Acute Myeloid Leukemia (AML) (NCT04429191) [16, 17] to Fanconi Anemia (FA) (NCT04784052) [18] have also shown promising results, however genotoxic agents, such as total body irradiation (TBI) or cyclophosphamide, have still been incorporated into these briquilimab-containing conditioning regimens [16–18].

Despite these exciting advances with αCD117 mAb-based conditioning approaches that are already decreasing toxicity of HSCT in certain patients, it is not fully known how these agents affect HSCs or why there is varying efficacy across disease settings. Further, due to lack of understanding of the mechanism of αCD117 mAb action and differing effects on HSCs in different disease settings, it is difficult to predict the efficacy of such mAb treatment without expensive and time-consuming disease-specific preclinical studies. Specifically, the α-mouse(m)CD117 mAb ACK2 has been shown to be an effective stand-alone conditioning agent to deplete host HSCs and successfully enhance donor chimerism post HSCT in certain disease settings, such as in SCID [11] and in-utero settings [19]. However, no efficacy has been found as a stand-alone agent in chronic granulomatous disease (CGD) [20], mucopolysacharidosis II (MPS II) [21], or unperturbed wild-type (WT) [20, 22] settings with conflicting results in the Fanconi Anemia setting [23, 24]. These unanticipated and unexplained differences of mAb-based conditioning in different SCID patients and in different disease mouse models complicate development of α-human(h)CD117 mAb-based conditioning for clinical use. Moreover, the lack of insight into the mechanism of αCD117 mAb-based conditioning and previous reports showcasing reliance on antibody-dependent cellular cytotoxicity (ADCC) [22] has slowed development of improved approaches that could enable completely non-toxic conditioning across all disease settings.

Given the potential impact of non-genotoxic mAb-based conditioning, we performed rigorous and novel experiments to understand αCD117 mAb conditioning in SCID and WT mouse models. We hypothesized that antagonism of SCF is sufficient for HSC depletion in SCID settings and that αCD117 mAbs with functional Fc are required for combined efficacy with αCD47 mAbs to enable use in WT settings. Moreover, we hypothesized that this may be the most effective non-genotoxic combination approach to enable high donor engraftment post HSCT in WT settings. To investigate these hypotheses, we first compared different α-mCD117 clones to understand their competitive binding to CD117 and ability to block SCF binding. Subsequently, we compared the capacity of these various clones to inhibit HSC proliferation in vitro, deplete HSCs in vivo in SCID mice and enable conditioning efficacy post HSCT in SCID mice as we have been previously shown with the ACK2 mAb [11]. We next generated various SCF-blocking fragment derivatives of the ACK2 mAb and similarly interrogated HSC depletion capacity in each of these settings. To enable efficacy in immunocompetent WT settings, we also explored combination approaches with αCD117 mAbs. While previous reports have documented combination ACK2 and αCD47 efficacy in the past [22], we additionally explored efficacy of other αCD117 mAbs as well as deglycosylated ACK2. Moreover, we also investigated additional αCD117 mAb combinations. Through these experiments we enable new understanding into the mechanism behind HSC depletion using CD117-targeting mAbs and efficacy of combination approaches. Insights learned from this research may pave the way for the development of clinical strategies that could enable non-genotoxic HSCT for a wide range of hematopoietic disorders affecting millions of individuals worldwide.

## Methods

### Mice

All animal procedures were approved by the International Animal Care and Use Committee (IACUC) at Stanford University. *B6.Cg-Rag2*^*tm1.1Cgn*^*/J* (SCID) mice were purchased from Jackson Laboratory (Bar Harbor, ME) and bred in house at Stanford University for all SCID experiments. Wildtype C57BL/6N (WT CD45.2) and B6.SJL-*Ptprc*^*a*^*Pepc*^*b*^/Boy (WT CD45.1) mice were purchased from Charles River Laboratories (Wilmington, MA). WT CD45.2 mice were used as recipients for all WT studies. WT CD45.1 mice were used as donors for all transplantation experiments, except those in Supplemental Fig. 8. C57BL/6N-CD45.1^STEM^ (WT CD45.1^STEM^) mice [25] were a generous gift from Prof. David Scadden at Massachusetts General Hospital/Harvard University and were used as donors for the combination study containing αCD110 mAb and hypomethylating agents. All animals were 8–12 weeks old at time of initiation of each study. All experiments were reported in line with the ARRIVE guidelines 2.0 and all no human cells was used in this research.

### Growth kinetics of mouse HSCs

In vitro studies using mouse HSCs were performed to assess HSC growth post exposure to αCD117 mAbs and fragments. Briefly, 10 mouse HSCs were sorted into individual wells of a 96-well round-bottom plate (See Supplemental Methods). Each well contained 100uL of HSC growth media consisting of F12 media supplemented with 10 mM HEPES, 1 × P/S/G, 1X ITSX, 1 mg/mL PVA, 100 ng/mL TPO, and 10 ng/mL SCF as previously reported [26]. Sorted HSCs were then treated with 100ug/mL of αCD117 mAbs or αCD117 mAb ACK2 fragments (See Supplemental Methods). Cells were cultured for 4 days and viable cells were subsequently counted under light microscopy. In addition, cells were collected and analyzed with counting beads (ThermoFisher, Waltham, MA) to verify cell counts by flow cytometry (See Supplemental Methods).

### Antibody treatment

All antibodies were purchased from Bio X Cell (Lebanon, NH) and diluted in sterile Phosphate-buffered saline (PBS), except αCD110 mAb which was obtained from Immuno-Biological Laboratories, Inc. (Minneapolis, MN). Control IgG was obtained from ThermoFisher (Waltham, MA). For dosing and injection information, see Supplemental Methods for details.

### Hematopoietic stem cell transplantation (HSCT)

For SCID recipient experiments, bone marrow (BM) cells were harvested from donor mice (see Supplemental Methods) and enriched for HSPCs with anti-mCD117 microbeads (Miltenyi, Auburn, CA) similar to prior reports [11]. Four million HSPCs were transplanted intravenously via retro-orbital (RO) injection into each SCID recipient mouse conditioned with αCD117 mAb or fragments at time of clearance (Supplemental Table 2) which was determined as previously reported [11]. For WT recipient experiments, BM cells were identically harvested from donor mice and 10 million WBM cells were similarly transplanted intravenously via RO injection under isoflurane anesthesia into each of the conditioned recipient mice.

### Assessment of in vivo HSC depletion and donor engraftment post HSCT

To evaluate for HSC depletion after treatment with αCD117 mAbs and the various additional agents, BM aspirate (BMA) samples [27] were acquired under isoflurane anesthesia immediately prior to HSCT. Samples were processed, stained using fluorescently labeled antibodies and analyzed via flow cytometry as previously reported [11, 24] (See Supplemental Methods for FACS analysis). For assessment of donor chimerism, peripheral blood (PB) samples were collected retro-orbitally into PBS with 0.5 mM EDTA every 4 weeks. At indicated time points post HSCT, necropsy was also performed to evaluate for donor chimerism in BM HSC. Samples were similarly processed, stained and analyzed by flow cytometry as previously reported [11, 24].

### Colony forming capacity (CFC) assessment

Mouse BM cells were obtained by aspiration or necropsy as indicated above. After removal of RBCs with RBC lysis buffer (eBioscience, San Diego, CA), cells were plated in triplicate for methylcellulose culture in Methocult M3434 (StemCell Technologies, Vancouver, Canada) as previously reported [11, 24]. Colony formation potential was evaluated 7 days post culture using the automated StemVision (StemCell Technologies) and recorded accordingly.

## Results

### αCD117 mAb ACK2, but not 2B8 or 3C11, uniquely blocks SCF binding to mCD117

**Fig. 1:**
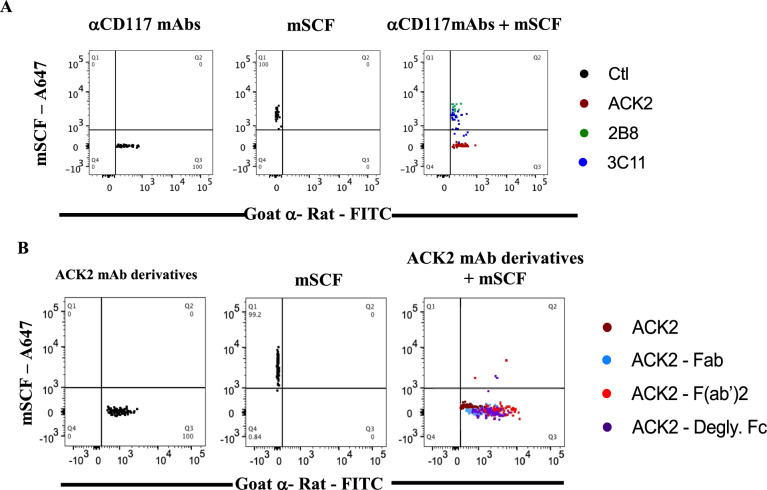
αCD117 ACK2 mAb has the unique property of inhibiting SCF binding which is maintained with Fab, F(ab’)2 and deglycosylated mAb derivatives. Representative flow cytometry plots showing the binding of αCD117 mAb to mouse HSCs (Lin^−^Sca-1^+^CD150^+^CD48^−^CD244^−^) detected by goat-α-Rat-FITC (left), fluorophore labeled mSCF-A647 (center), and various αCD117 mAbs followed by mSCF-AF647 (right) with overlayed graphs of **A** untreated control (Ctl) and αCD117 mAbs (clone ACK2, 2B8, and 3C11). **B** αCD117 ACK2 mAb compared to ACK2 fragments Fab, F(ab’)2, and mAb with deglycosylated Fc were subsequently similarly tested for SCF-AF467 binding antagonistic capacities (n = 5–7)

To explore the role of SCF-blockade on HSCs, we first sought to identify αCD117 mAbs with differing binding properties to mouse CD117. These were tested for binding and blockade of SCF on a c-kit^+^ P815 mouse mast cell line (Supplemental Table 1). Next, HSCs isolated from mouse BM were treated with three different αCD117 mAb clones ACK2, 2B8, and 3C11, and fluorochrome conjugated mSCF was added to antibody pre-coated HSCs to test for blockade. ACK2 pre-coated HSCs showed antagonism of SCF with complete inhibition of its binding to HSCs (Fig. 1A). Conversely, SCF similarly bound to the untreated control and 2B8 pre-coated HSCs, and we detected partial binding of SCF to 3C11 pre-coated HSCs (Fig. 1A).

To further characterize the mechanism behind ACK2’s efficacy and to interrogate the impact of SCF blockade, we generated αCD117 mAb ACK2 fragments including Fab and F(ab’)2. Additionally, we generated ACK2 with a deglycosylated fragment crystallizable region (Fc) that does not have active Fc effector function for immune system activation (Supplemental Fig. 1A–C). We found that ACK2 mAb fragments and the deglycosylated mAb all exhibited identical antagonistic properties as ACK2 and inhibited binding of SCF on HSCs to similar levels (Fig. 1B). These results suggest that αCD117 mAb ACK2 and mSCF interact through antagonistic blockade and likely share a binding epitope on c-KIT, consequently resulting in inhibited binding of SCF on HSCs.

### αCD117 mAb ACK2 and its fragment derivatives inhibit HSPC proliferation in vitro

**Fig. 2:**
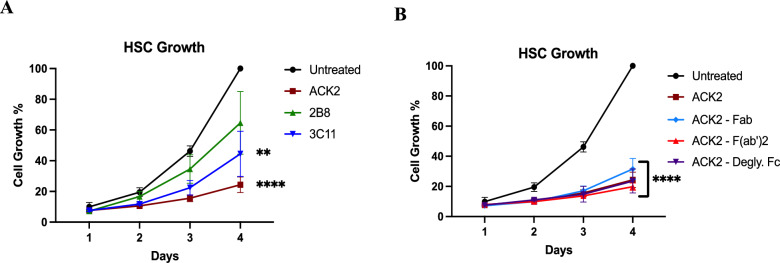
Only αCD117 ACK2 mAb and its derivatives robustly inhibit HSC growth and differentiation in vitro. FACS sorted mouse HSCs (Lin^−^Sca-1^+^CD150^+^CD48^−^CD244^−^) were cultured for 4 days with **A** different αCD117 mAb clones and **B** ACK2 mAb derivatives (Fab, F(ab’)2, and mAb with deglycosylated Fc) to determine effects on HSPC growth kinetics as assessed by light microscopy (n = 5–7). Statistics calculated using unpaired t-test compared with untreated controls (*P < 0.05; **P < 0.01; ***P < 0.001; ****P < 0.0001)

Next, we explored the effects of SCF blockade on HSPC proliferation using αCD117 mAbs and their derivatives. Growth kinetics from HSCs (Lin^−^Sca-1^+^CD150^+^CD48^−^CD244^−^) were measured in the presence of αCD117 mAbs and fragments in vitro. Only ACK2 inhibited proliferation of HSPCs compared to the other tested αCD117 mAbs (Fig. 2A). The 3C11 treated group achieved intermediate results between the ACK2 and 2B8 treated groups (Fig. 2A), which is likely explained from its partial antagonistic property of SCF. HSPC proliferation in the 2B8 treated group remained similar to the untreated group, which aligned with its non-antagonistic property to SCF (Fig. 2A). Importantly, similar results to the full ACK2 were also obtained with the ACK2 fragment derivatives with a profound reduction in the number of HSPCs post in vitro culture (Fig. 2B). These results highlight the unique inhibition of HSC proliferation in vitro caused by co-culture with any mAb derivative containing the ACK2 Fab region, and further highlight that at least one mechanism of ACK2-mediated HSC depletion relies on its SCF blockade and not Fc receptor mediated activity.

### SCF-blockade is required and sufficient to deplete HSCs in vivo and enhance engraftment post HSCT in SCID settings

**Fig. 3:**
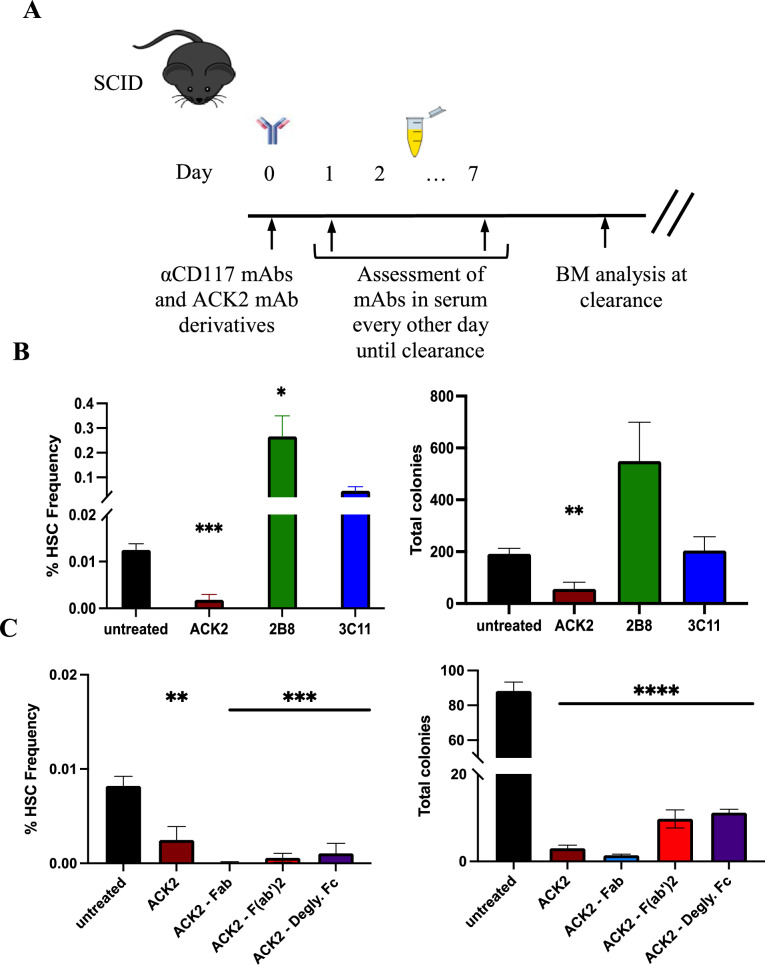
αCD117 ACK2 mAb and its fragment derivates uniquely deplete HSCs in SCID settings. **A** Experimental outline to assess the time of clearance of each αCD117 mAb (clone ACK2, 2B8, 3C11) or each αCD117 ACK2 mAb derivatives (Fab, F(ab’)2, and mAb with deglycosylated Fc) in the SCID mouse model with subsequent assessment of HSC depletion at time of mAb or fragment clearance. **B** BM HSC depletion was measured when αCD117 mAbs were cleared in peripheral blood (PB) serum and compared to untreated control mice. Profound BM HSC depletion was only observed post treatment with αCD117 ACK2 mAb as determined by phenotypic assessment with flow cytometry (Lin^−^Sca-1^+^CD117^+^CD150^+^CD48^−^) and functional assessment of HSPCs by in vitro colony forming capacity. (C) Subsequently, a parallel experiment was performed assessing αCD117 ACK2 mAb fragment derivatives’ depletion capacity with similarly robust HSC depletion observed in all treatment groups as determined by phenotypic assessment by flow cytometry (Lin^−^Sca-1^+^CD117^+^CD150^+^CD48^−^) and functional assessment by in vitro colony forming capacity. (n = 5). Statistics calculated using unpaired t-test compared with untreated controls (*P < 0.05; **P < 0.01; ***P < 0.001; ****P < 0.0001)

To determine if the antagonistic effect of αCD117 mAb ACK2 with SCF observed in vitro correlated with the in vivo HSC depletion and enhanced engraftment post HSCT that was previously documented [11], we investigated the effects of different αCD117 mAb variants in a SCID mouse model. Prior to HSCT, we measured the clearance of αCD117 mAbs in serum to determine the optimal time for HSCT that would not result in graft depletion (Fig. 3A, Supplemental Table 2). Each αCD117 mAb cleared at different rates, with the 2B8 mAb maintaining the longest persistence in the PB serum 15 days post treatment, compared to 9- and 11-days post treatment for ACK2 and 3C11 mAbs respectively (Supplemental Table 2). Clearance time of the ACK2 fragment derivatives was identically assessed. Due to the decreased half-life of the ACK2 mAb Fab and F(ab)’2 fragments, we increased the dose administered to 1.5 mg of each fragment treatment per animal to control the exposure time of HSCs to each agent similar to the ACK2 mAb. Even at this dose, the Fab and F(ab)’2 fragments cleared faster than the full ACK2 and deglycosylated Fc derivative at 3- and 5-days post treatment compared to 7–9 days post treatment respectively (Supplemental Table 2).

At time of serum clearance of each αCD117 mAb and fragment, BM was assessed by aspirate for phenotypic and functional HSC depletion (Fig. 3A). Only ACK2 achieved profound phenotypic HSC depletion as determine by flow cytometry confirming prior reports[11] (Fig. 3B). In the ACK2 treated group, effects on BM progenitors were also assessed which showcased additional depletion of myeloid progenitors (MyPro, MP), common myeloid progenitors (CMP), granulocyte-monocyte progenitors (GMP), megakaryocyte-erythrocyte progenitors (MEP), and common lymphoid progenitors (CLP) (Supplemental Fig. 2). Surprisingly, HSC frequency increased dramatically in 2B8 and 3C11 treated groups by more than a 20-fold and tenfold increase respectively (Fig. 3B). In contrast to prior published reports[22], the ACK2 fragment treated groups exhibited similar phenotypic HSC depletion (Fig. 3C). The degree of phenotypic HSC depletion in each mAb and fragment treated group also correlated with in vitro functional HSPC assessment, where the colony forming count (CFC) activity was decreased in the ACK2 and fragment treated groups, and increased in the 2B8 and 3C11 treated groups (Fig. 3B, C).

**Fig. 4:**
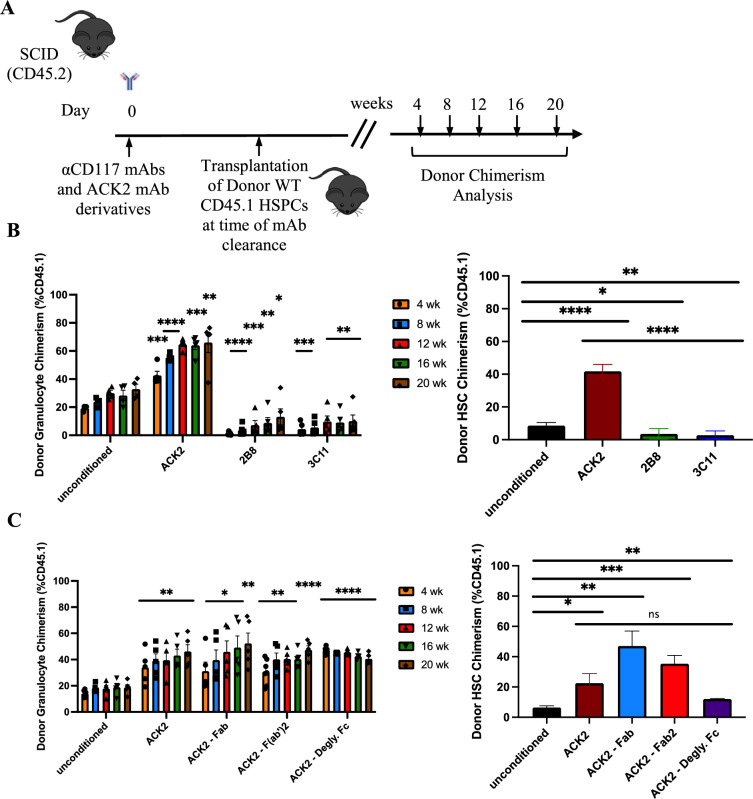
αCD117 ACK2 mAb and its fragment derivates uniquely enhance donor engraftment post HSCT in SCID settings. **A** Experimental outline to assess the conditioning efficacy of each αCD117 mAb (clone ACK2, 2B8, 3C11) or each αCD117 ACK2 mAb derivatives (Fab, F(ab’)2, and mAb with deglycosylated Fc) in the SCID mouse model with subsequent assessment of donor chimerism post HSCT. **B** PB donor chimerism and BM HSC donor chimerism were measured 20 weeks post HSCT of αCD117 mAb conditioned animals with 4 × 10^6^ CD45.1 donor cKIT^+^ enriched cells. Enhanced donor engraftment was only observed post conditioning with αCD117 ACK2 mAb, whereas αCD117 2B8 and 3C11 mAbs decreased donor engraftment (n = 5). **C** Subsequently, a parallel experiment was performed assessing αCD117 ACK2 mAb fragment derivatives’ conditioning capacity with similarly robust donor engraftment observed with full intact αCD117 ACK2 mAb and its Fab, F(ab’)2, and deglycosylated Fc derivative (n = 5). Statistics calculated using unpaired t-test compared with unconditioned controls (*P < 0.05; **P < 0.01; ***P < 0.001; ****P < 0.0001)

HSCT was subsequently performed in each mAb treated group at time of mAb clearance (Fig. 4A), and donor engraftment was not surprisingly found to correlate to the respective HSC depletion data before HSCT. Notably, both ACK2 and its derivative fragment treated groups significantly enhanced HSPC engraftment with equivalent high donor chimerism in granulocytes of > 50% and HSCs of > 30% 20 weeks post HSCT (Fig. 4B, C). In contrast, 2B8 and 3C11 mAb treated groups had the least donor chimerism 20 weeks post HSCT with ~ 10% in granulocytes and ~ 3% in HSCs (Fig. 4B). Our results emphasize the importance of the antagonistic property of the conditioning αCD117 mAb and its SCF-blocking derivatives which all enabled enhanced donor HSPC engraftment post HSCT in the SCID mouse model. Further, they specifically show that a functional Fc is not required for in vivo HSC depletion and effective conditioning in SCID settings given the effects observed with antagonistic mAb fragments and deglycosylated mAb.

### Functional Fc and SCF-blockade are required to enhance αCD117 mAb efficacy with αCD47 mAb combination in wildtype settings

**Fig. 5:**
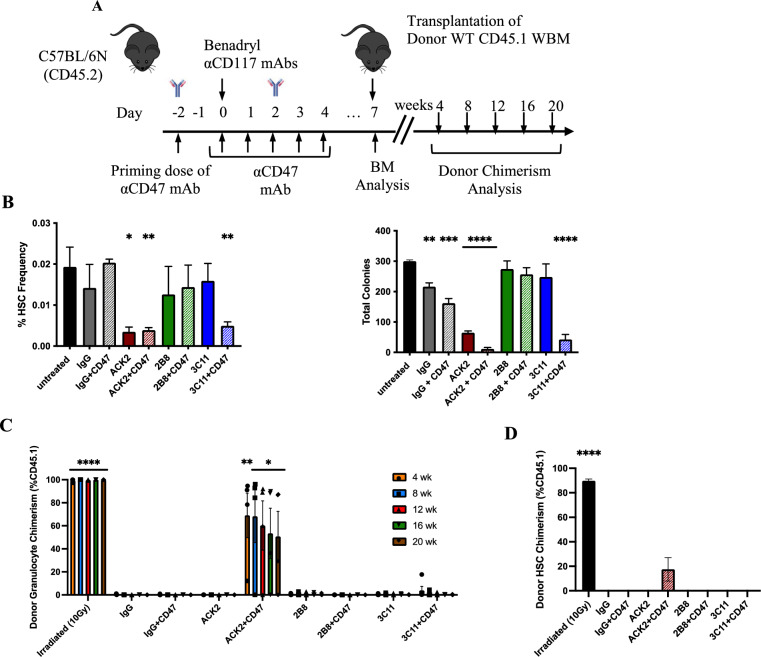
Only αCD117 ACK2 mAb augmented with αCD47 mAb enables depletion of HSCs and enhances donor engraftment post HSCT in WT settings. **A** Experimental outline to showcase mAb treatment and assess HSC depletion and HSCT transplantation efficacy with combination αCD117 mAbs and αCD47 mAb treatment in WT mice. **B** BM HSC depletion was measured 7 days post treatment with profound depletion in αCD117 ACK2 and 3C11 mAb augmented with αCD47 mAb treated groups as determined by phenotypic assessment by flow cytometry (Lin^-^Sca-1^+^CD117^+^CD150^+^CD48^−^) and functional assessment by *in vitro* colony forming capacity. **C** PB donor chimerism and **D** BM HSC donor chimerism were measured 20 weeks post HSCT of conditioned animals with 10 × 10^6^ CD45.1 donor WBM cells. Only αCD117 ACK2 mAb augmented with αCD47 mAb was observed to result in robust donor chimerism in peripheral blood and HSCs (n = 5). Statistics calculated using unpaired t-test compared with unconditioned controls (*P < 0.05; **P < 0.01; ***P < 0.001; ****P < 0.0001).

Recent studies have shown that HSCT conditioning can be achieved in WT settings with αCD117 mAb ACK2 augmented with a blocking αCD47 mAb [22]. αCD47 mAbs have been found to increase phagocytosis of cells with pro-phagocytosis signals which can be intrinsic to cells or due to mAb binding. Given the different levels of antagonism that we identified with the αCD117 mAbs in vitro, we sought to determine how this correlated to HSC depletion capacity and HSCT conditioning efficacy of each αCD117 mAb augmented with αCD47 mAb. Mice were treated with both agents and BM depletion was analyzed 7 days after combination antibody treatment was completed (Fig. 5A). Only antagonistic ACK2 + CD47 mAb and partially antagonistic 3C11 + CD47 mAb treated groups exhibited significantly decreased HSC frequency post treatment (Fig. 5B). In addition, HSPC function as assessed via in vitro CFC assay also showed profound decline of colony forming potential only in the ACK2 + CD47 mAb group with decreased but more limited decline in the 3C11 + CD47 mAb group (Fig. 5B). BM hypocellularity was also observed on histology of femurs in ACK2 + CD47 mAb and 3C11 + CD47 mAb treated groups as compared to untreated or αCD117 mAb alone treated groups (Supplemental Fig. 3). In addition, BM progenitors including MP, CMP, GMP, MEP, and CLP were also significantly decreased in ACK2 and ACK2 + CD47 mAb treated groups in the WT mice (Supplemental Fig. 4). Combination ACK2 + CD47 mAb additionally led to significantly decreased white blood cells (WBC), red blood cells (RBC), and hematocrit (HCT) compared to ACK2 mAb conditioned alone though no decrease in platelets was observed (Supplemental Fig. 5A). No other toxicities were found in the spleen or the liver, though histology assessment showed apoptosis of various hematopoietic precursor cells 7 days post ACK2 + CD47 mAb treatment (Supplemental Fig. 5B–D).

Donor engraftment post combination mAb-based conditioning and HSCT grossly correlated to the degree of HSC depletion by phenotypic and functional assessment before HSCT (Fig. 5C, D). As previously reported [22], the ACK2 + CD47 mAb conditioned group developed and maintained long-term donor chimerism in PB and BM HSC 20 weeks post HSCT. However, no robust long-term engraftment was noted after HSCT with either alternative αCD117 mAb and CD47 mAb combination conditioning. Although a small amount of donor chimerism was initially observed at early time points in the 3C11 + CD47 mAb treated group which may have been due to the partial SCF antagonism of the 3C11 mAb, it only demonstrated short-term engraftment from transplanted graft progenitors without the ability to develop into long-term donor chimerism which was surprising given the extent of HSC depletion and BM depletion. Potentially the initial donor engraftment in this group may have not been sufficient to overcome the minor allelic mismatches between the WT donor CD45.1 and recipient CD45.2 mice [28, 29] or alternatively there may have been depletion of the donor HSCs in the graft from delayed mAb clearance.

**Fig. 6:**
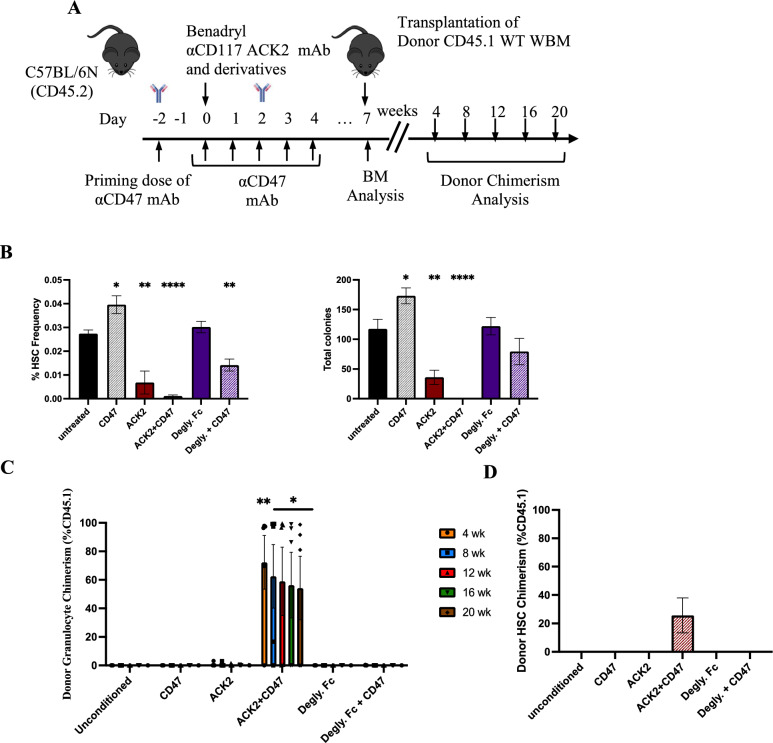
Functional Fc is required for αCD117 ACK2 mAb and αCD47 mAb combination treatment to results in HSC depletion and enhanced donor engraftment post HSCT in WT settings. **A** Experimental outline to assess mAb treatment, HSC depletion and HSCT transplantation efficacy with combination αCD117 ACK2 mAb, deglycosylated Fc derivative and αCD47 mAb treatment in WT mice. **B** BM HSCs depletion was measured 7 days post treatment with profound depletion only observed post intact αCD117 ACK2 mAb and αCD47 mAb treatment as determined by phenotypic assessment by flow cytometry (Lin^-^Sca-1^+^CD117^+^CD150^+^CD48^-^) and functional assessment by *in vitro* colony forming capacity. **C** PB donor chimerism and **D** BM HSC donor chimerism were measured 20 weeks post HSCT of conditioned animals with 10 × 10^6^ CD45.1 donor WBM cells with robust donor engraftment only observed with conditioning with the full αCD117 ACK2 augmented with αCD47 mAb (n = 5). Statistics calculated using unpaired t-test compared with unconditioned controls (*P < 0.05; **P < 0.01; ***P < 0.001; ****P < 0.0001).

As we found that αCD117 mAb fragments maintained similar HSC depletion and conditioning activity compared as αCD117 mAb ACK2 in the SCID setting, we were interested to determine the ability of the fragments to complement αCD47 mAb activity and to determine if the Fc region was necessary for functionality. We focused our attention on the deglycosylated Fc ACK2 mAb derivative due the immediate translational potential of this combination approach given that both an aglycosylated antagonistic αCD117 mAb, briquilimab, and an inhibitory αCD47 mAb, magrolimab, are being concurrently tested in clinical trials [14, 15, 18, 30]. The timeline for antibody administration for αCD117 mAb and αCD47 mAb remained similar with a priming dose of αCD47 mAb and transplantation 7 days after the first administration of the full dose of αCD47 mAb (Fig. 6A). Unfortunately, phenotypic and functional HSC depletion was not as profound in the deglycosylated mAb group compared to full ACK2 + CD47 mAb treated group (Fig. 6B). Similarly, increased donor engraftment post HSCT was only achieved in ACK2 + CD47 mAb treated group as determined by both donor PB and BM HSC chimerism 20 weeks post HSCT (Fig. 6C, D). Our result show that it is essential to have immune system engagement for naked αCD117 mAb-based conditioning to be effective in the WT setting, and moreover we show that both antagonistic mAb and functional Fc are necessary for synergy with αCD47 mAbs. Taken together, these results explain the mechanism of action of ACK2 with and without αCD47 mAbs and with or without functional Fc receptor are different in various immunocompetency settings.

### Alternative combinations with antagonistic αCD117 mAbs do not result in efficient HSCT conditioning apart from hypomethylating agents

In addition to αCD47 mAb augmentation of αCD117 mAbs, we attempted to increase the efficacy of αCD117 mAb HSC depletion in WT mice with different HSC perturbation approaches including mobilization with G-CSF and plerixafor, branched-chain amino acid (BCAA)-free diet which specifically lacks Valine that has been shown to be critical for HSCs [31], antagonistic anti- anti-CD110 mAb, and hypomethylating agents azacytidine and decitabine.

We first examined the effects of G-CSF and/or plerixafor on HSCs in the PB and BM before HSCT using standard dosing that has been previously reported [32, 33] (Supplemental Fig. 6A). Alone and in combination with antagonistic αCD117 mAb ACK2, HSCs were effectively mobilized into the PB with each regimen (Supplemental Fig. 6B, C). Additionally, we observed an increase in HSCs in the BM with mobilization alone and a slight decrease with the mobilization + ACK2 mAb combinations (Supplemental Fig. 6B, C). The donor chimerism post HSCT reflected the HSC depletion data with no long-term donor chimerism in PB and HSC detected in any groups treated with the mobilization reagents alone unlike the irradiation control (Supplemental Fig. 6D, E). Specifically, while the mobilization + ACK2 mAb combinations showed short-term donor PB engraftment 4 weeks post HSCT, no long term engraftment at 20 weeks post HSCT was observed (Supplemental Fig. 6D, E).

Likewise, a BCAA-free diet was also explored in combination with antagonistic αCD117 mAb ACK2 (Supplemental Fig. 7A). Despite a robust decrease in the frequency of phenotypic HSCs and functional HSPCs in the combination ACK2 + BCAA-valine free diet group before HSCT (Supplemental Fig. 7B), no long-term donor chimerism was detected in PB or BM HSCs for all treated groups 20 weeks post HSCT (Supplemental Fig. 7C, D). While it is unknown why the lack of efficacy in this setting despite the robust HSC depletion, maintaining a healthy microenvironment in the BM without depriving essential nutrients also may be critical for donor HSC engraftment. As with the 3C11 + CD47 mAb treatment, it is also possible that the initial donor engraftment with these treatments may have been insufficient to overcome the minor allelic mismatches between the WT CD45.1 and CD45.2 mice.

Lastly, we also explored additional antibody and hypomethylating combinations to explore alternatives to αCD117 + αCD47 mAb combinations (Supplemental  Fig. 8A). Specifically, repeat doses of antagonistic αCD117 mAb ACK2 and an antagonistic αCD117 + αCD110 mAb combination was investigated, as was antagonistic αCD117 mAb ACK2 with hypomethylating agents azacytidine and decitabine. Robust phenotypic HSC depletion in the BM of treated mice was only observed with the antagonistic αCD117 + αCD110 mAb combination and the hypomethylating agents with and without ACK2 (Supplemental  Fig. 8B). However, only a slight increase in donor engraftment post HSCT in the antagonistic αCD117 + αCD110 mAb combination was observed with this dosing regimen (Supplemental Fig. 8C). In contrast, high donor engraftment was observed post HSCT with the ACK2 + azacytidine combination conditioning as was previously reported [34] and even higher engraftment was observed post HSCT with ACK2 + decitabine combination conditioning (Supplemental Fig. 8D).

## Discussion

Despite recent advances in the development of non-genotoxic antibody-based conditioning, genotoxic chemotherapy and/or irradiation still remain standard conditioning agents used to enable HSCT which have both significant short-term and long-term side effects [2]. Though various pre-clinical non-genotoxic conditioning approaches have now emerged including naked antibodies [11–24], antibody–drug-conjugates (ADCs) [35–40] and chimeric antigen receptor (CAR) T-cells [41–45] aimed now at various HSC targets (including but not limited to CD45, CD110, CD117 and CD123), only naked αCD117 mAbs have shown safety in patients to date [14–18] and toxicity concerns have either halted or slowed translation of ADC and CAR T-cell based approaches. Given this, in this study, we investigated the properties of “naked” (or unconjugated) αCD117 mAbs and their in vitro and in vivo effects on HSCs in SCID and WT mice to better understand the role of each functional component of the mAb in order to optimize translation of this conditioning approach to patients.

We have confirmed that the originally reported αCD117 conditioning mAb ACK2 possesses a unique antagonistic property inhibiting binding of the CD117 ligand SCF [10] and found that this correlates with the previously reported profound effects on HSCs in vitro and in vivo[11] which was not seen with the non-antagonistic αCD117 mAbs. Notably, we found that αCD117 mAb ACK2, and its SCF-blocking derivatives (Fab, F(ab)’2, and deglycosylated Fc mAb), similarly suppressed HSC growth in vitro and enabled robust depletion of HSCs in vivo in SCID mice which resulted in enhanced donor chimerism post HSCT. This conflicts with prior reports that ACK2 Fab lacks HSC depletion capacity [22]. Interestingly, treatment with non-antagonistic αCD117 mAb expanded HSCs in the BM and decreased donor engraftment post HSCT in the SCID setting. The results from the fragment treated groups confirmed our hypothesis that SCF blockade directly effects HSC growth and enhances donor engraftment post HSCT. This finding further supports that HSC depletion in SCID settings does not require a functional Fc region which mediates complement-dependent cellular phagocytosis (CDCP), antibody-dependent cellular cytotoxicity (ADCC), antibody-dependent phagocytosis (ADCP) or complement-dependent cytotoxicity (CDC) [46]. As antibody fragments can retain similar efficacy to the full length mAb, it is maybe useful to develop small antibody fragments for therapeutic purposes given their shorter half-life. This may be advantageous for use as conditioning agents to reduce waiting time for antibody clearance and be able to proceed to HSCT without concern about circulating mAb-mediated depletion of donor HSCs from the graft.

However, as previously reported, SCF blockade alone was not sufficiently potent to deplete HSCs or enhance donor engraftment post HSCT in the WT setting [20, 22]. This is especially important to note as various groups are now developing CD117 epitope editing systems to protect edited HSCs from antagonistic αCD117 mAbs [45, 47], though to date most efforts have been focused on in vitro protection or protection in vivo in immunodeficient SCID mouse models where antagonistic αCD117 mAb efficacy is more robust than in WT immunocompetent settings. Similar studies have yet to be reported in immunocompetent settings using epitope editing of mouse or NHP HSCs to assess for selective advantage under antagonistic αCD117 mAb selection pressure. Although it is unknown why antagonistic αCD117 mAbs and fragments effectively deplete HSCs in SCID and not WT immunocompetent settings, we have recently demonstrated that the αCD117 mAb ACK2 disrupts the MAPK pathway [48]. This suggests that the microenvironment is different between these two settings and that this blockade is uniquely sufficient in SCID settings.

Further, while it has been previously reported that efficacy can be augmented with combination αCD117 and αCD47 mAbs [22], we determined from this work that this likely requires an antagonistic αCD117 mAb with a competent antibody Fc region for immune system activation to enhance the HSCT outcome in the WT setting as no long-term donor chimerism was detected when animals were co-treated with other αCD117 mAbs or with the ACK2 deglycosylated Fc derivative. Given that the most advanced αCD117 conditioning mAb, briquilimab, derived from our efforts is an unconjugated, aglycosylyated antagonistic mAb [13–15], this work emphasizes the likely need for development of new αCD117 mAbs with active Fc if enhanced potency in combination with clinical αCD47 mAbs for ADCC is desired. Further, it suggests that these αCD117 mAbs should be antagonistic to CD117 for most potent combination activity with αCD47 mAbs. No other combination explored resulted in similarly robust conditioning efficacy apart from combinations with genotoxic hypomethylating agents. Alternatively, based upon on our past findings non-antagonistic αCD117-ADCs may be an alternative approach for efficacy across settings if toxicity can be overcome [36–39, 49–51].

## Conclusion

Given the large need for safer HSCT approaches and the recent advances in αCD117 mAb conditioning agents that are now in development by over a dozen groups, we believe understanding the mechanism of αCD117 mAbs on HSCs and within HSCT conditioning to be of critical importance. While this work sheds additional light on these mechanisms, much remains to be learned about the science of conditioning. Through additional research on the effects of conditioning, we aspire to develop improved approaches that enable completely safe and effective HSCT across all settings enabling this powerful curative treatment to be used more efficiently in the millions of patients worldwide that currently suffer from diverse blood and immune diseases.

## Supplementary Information

Supplementary file 1 (PDF 2634 kb)

## Data Availability

Data that support the findings of this study are available from the corresponding author upon reasonable request.
