# Supplementary material for: Targeted hematopoietic stem cell depletion through SCF-blockade
Source: Stem Cell Res Ther. 2024 Oct 29;15:387. doi: 10.1186/s13287-024-03981-0 (PMC11523590; doi:10.1186/s13287-024-03981-0)
Supplement: Supplementary file 1 — (PDF 2634 kb) [file 13287_2024_3981_MOESM1_ESM.pdf]

SUPPLEMENTAL MATERIALS

METHODS CONTINUED

Necropsy, bone marrow isolation and bone marrow analysis

Mice were euthanized by carbon dioxide asphyxiation, and the surface of the skin was disinfected with 70% alcohol. The lower limbs of the mice were removed with scissors, and the muscles and soft tissues attached to the bones were removed, leaving clean, bilateral femurs and tibias. Bones were crushed in 1X PBS supplemented with 2% serum using a mortar and pestle and then passed through a 70um cell strainer to remove debris as previously reported [11][24]. BM from donor mice was used for *in vitro* experiments or for transplantation studies as further detailed in the methods. BM from recipient mice was analyzed by flow cytometry. Blood counts and histology with hematoxylin and eosin (H+E) staining were performed by the Stanford Veterinary laboratory.

Generation of αCD117 mAb ACK2 fragments

αCD117 mAb ACK2 fragments Fab, F(ab)'2, and deglycosylated Fc derivative were generated using Pierce Fab preparation kit (ThermoFisher, Waltham, MA), F(ab')2 preparation kit (ThermoFisher), and Immobilized GlycINATOR (Genovis, Lund, Sweden) respectively according to the manufacturer's instructions. ACK2 mAb fragments were purified using Amicon Ultra-4 Centrifugal Filter Unit (Sigma-Aldrich) with manufacturer's instructions. SDS-PAGE was performed to confirm the size of each fragment.

Assessment of SCF epitope binding and SCF blockade on c-kit^{+} cell line

mSCF was conjugated with Alexa Fluor 647 using antibody labeling kit (ThermoFisher, Waltham, MA) according to the manufacturer's instructions. αCD117 mAbs and mAb fragments were incubated with serum and cytokine depleted mouse c-KIT^{+} P815 mast cell line (ATCC, Manassas, VA) for epitope competition and SCF blockade studies. Subsequently a single cell suspension was stained with mSCF-AF647 and followed by goat-anti-rat FITC, a secondary antibody for αCD117mAbs and fragments. Results were analyzed by flow cytometry assessing for A647 and FITC fluorescence.

Isolation and assessment of SCF blockade on mouse HSCs

Mouse BM cells were isolated from WT CD45.2 mice and first lineage-depleted using magnetic beads (Miltenyi, Auburn, CA). HSCs were subsequently isolated using FACS on a BD FACSAria II based upon Lin-Sca-1+CD150+CD48-CD244- expression. cKIT surface marker was not included for phenotypic identification of HSCs to minimize confounding results of competing αCD117 mAbs bound to the cell surface. Rather, the CD244 surface marker was used as we aimed to analyze primitive HSCs without using CD117 [49]. Subsequently, HSC were treated with αCD117 mAb clones ACK2, 2B8, and 3C11 or ACK2 fragments, and fluorochrome conjugated mSCF was similarly added to antibody pre-coated HSCs to test for blockade as with the P815 mast cells.

Antibody treatment continued

To prevent mast cell degranulation, 2mg/mL of diphenhydramine (Sigma-Aldrich, St. Louis, MO) was injected intraperitoneally (IP) 15 minutes prior to injection of conditioning antibodies (see methods). 500ug of αCD117 mAbs (clone ACK2, 2B8, and 3C11), 1.5mg of αCD117 mAb ACK2 Fab and F(ab)'2 fragment, and 500ug of αCD117 mAb ACK2 deglycosylated Fc derivative were each administered intravenously (IV) via retro-orbital injection as per previous reports [11]. For combination studies with αCD47 mAb, 100ug of αCD47 mAb (MIAP410) was administered as a priming dose on day -2 IP and subsequently 500ug was administered daily for 5 consecutive days to enhance mAb efficacy and HSC depletion [22]. For combination studies utilizing αCD110, clone AMM2 was administered IV 7 days prior to HSCT at 5mg/kg per dose via retro-orbital injection. Anesthesia was utilized for all retro-orbital injections, transplants and bone marrow aspirated. Animals were anesthetized using Isoflurane MWI Animal Health, Shakopee, MN) via inhalation. Mice were induced at 3-4% isoflurane and maintained at 1-2%.

HSPC mobilization, administration of BCAA-valine free diet and hypomethylating agent treatment

Following previously published protocols, GCSF (Amgen, Thousand Oaks, CA) was administered twice per day at 6.25ug per dose subcutaneously (SQ) for 4 consecutive days [32], followed by HSCT or treatment with αCD117 mAb ACK2 7 days before HSCT. 5mg/kg of plerixafor (Genzyme Corporation, Cambridge, MA) was given SQ 1 hour before HSCT or treatment with αCD117 mAb ACK2 7 days before HSCT [33]. In a separate experiment, animals were fasted for 48 hours before being given a Branched-chain amino acid (BCAA)-valine free diet for 14 days until HSCT [31], gift from Nakauchi lab which originated from Research Diets Inc. 1 week before HSCT, animals were treated with αCD117 mAb ACK2 to augmented the BCAA-valine free diet. For combination studies utilizing hypomethylating agents, either azacytidine or decitabine was used. Azacytidine (Sigma-Aldrich, St. Louis, MO) was administered once per day at 5mg/kg per dose intraperitoneally (IP) for 5 consecutive days, followed by HSCT [34]. Decitabine (Sigma-Aldrich, St. Louis, MO) was administered once per day at 1mg/kg per dose IP for 5 consecutive days [50], followed by HSCT.

FACS analysis

All samples were acquired using a BD FACSAria II flow cytometer and analyzed using FlowJo software. Gating strategy was defined as follows: B-cells (Ter119^{-}CD3^{-}B220^{+}CD19^{+}), Cytotoxic T-cells (Ter119^{-}CD3^{+}B220^{-}CD8^{+}CD4^{-}), Helper T-cells (Ter119^{-}CD3^{+}B220^{-}CD8^{+}CD4^{+}), and Granulocytes (Ter119^{-}CD3^{-}B220^{-}CD11b^{+}Gr-1^{+}), Bone marrow HSCs (Lin^{-}Sca-1^{+}CD117^{+}CD150^{+}CD48^{-}), Myeloid progenitors (MyPro,MP) (Lin^{-}Sca-1^{-}CD117^{+}IL7ra^{-}), Common Myeloid Progenitors (CMP) (Lin^{-}Sca-1^{-}CD117^{+}IL7ra^{-}CD34^{+}CD16/32^{-}), Granulocyte-monocyte Progenitors (GMP) (Lin^{-}Sca-1^{-}CD117^{+}IL7ra^{-}CD34^{+}CD16/32^{+}), Megakaryocyte-Erythrocyte Progenitors (MEP) (Lin^{-}Sca-1^{-}CD117^{+}IL7ra^{-}CD34^{-}CD16/32^{-}), and Common Lymphoid Progenitors (CLP) (Lin^{-}Sca-1^{-}CD117^{+}IL7ra^{+}) [51].

Statistics

All experimental data was analyzed using GraphPad Prism version 10.2.2 and was analyzed with unpaired t-tests and non-parametric tests for significance (∗) P<0.05; (∗∗) P<0.01; (∗∗∗) P<0.001; (∗∗∗∗) P<0.0001. The statistical data are presented as standard error of the mean (SEM).

Supplemental Table 1

		Blocking						
**Precoated Antibody**	**Isotype**	**pSCF (ligand)**	**mSCF (ligand)**	**ACK2**	**ACK45**	**ACK4**	**2B8**	**3C11**
**ACK2**	rat IgG2b, κ-1a	+	+		+	-	-	-/+
**ACK45**	rat IgG2b, κ	+	ND	+		-	ND	ND
**ACK4**	rat IgG2a	-	ND	ND	ND		ND	ND
**2B8**	rat IgG2b, κ	-	-	-/+	-	+		-/+
**3C11**	rat IgG2b, κ	-	-/+	-	-	-	-	

Supplemental Table 1: Epitope mapping of αCD117 mAbs and SCF blockade assessment on c-kit$^+$ cell line. Summary table of epitope blocking for various αCD117 mAbs and SCF on murine P815 mast cells. αCD117 ACK2 mAb displayed full blockade of porcine-SCF (pSCF) and mouse-SCF (mSCF), whereas other αCD117 mAb clones either do not block or partially block pSCF and mSCF. (+; +/-; -; ND: defines as antagonistic, partially antagonistic, non-antagonistic, and not determined respectively).

Supplemental Table 2

	**Dose**	**Day of Clearance**
**ACK2**	500ug	Day 9
**2B8**	500ug	Day 15
**3C11**	500ug	Day 11
**ACK2 - Fab**	1.5mg	Day 3
**ACK2 – F(ab)'2**	1.5mg	Day 5
**ACK2 – Deglycosylated Fc**	500ug	Day 7

Supplemental Table 2: αCD117 mAbs have different clearance rates in SCID mice. Summary Table of αCD117 mAb clearance in SCID mice. Higher amount of αCD117 ACK2 mAb fragment Fab and F(ab')2 were used due to more rapid clearance. Different serum clearance rates were observed ranging from 3 to 15 days post treatment of αCD117 mAbs (n=5).

Supplemental Figure 1

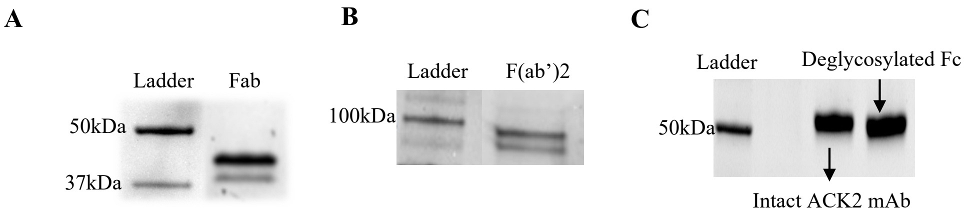

Supplemental Figure 1: Successful generation of αCD117 ACK2 mAb derivatives. αCD117 ACK2 mAb derivatives were generated using fragment preparation kit with Papain (Fab), Pepsin (F(ab')2), and Glycinator Enzyme (Deglycosylated Fc). The fragments were quality controlled by electrophoresis with SDS-PAGE. The **(A)** size of Fab fragment was 45-50kDa and **(B)** 100-110kDa for F(ab')2 fragment. **(C)** Deglycosylated Fc fragment has heavy chain that is 4kDa less than the heavy chain of intact ACK2, approximately with the size of 50kDa.

Supplemental Figure 2

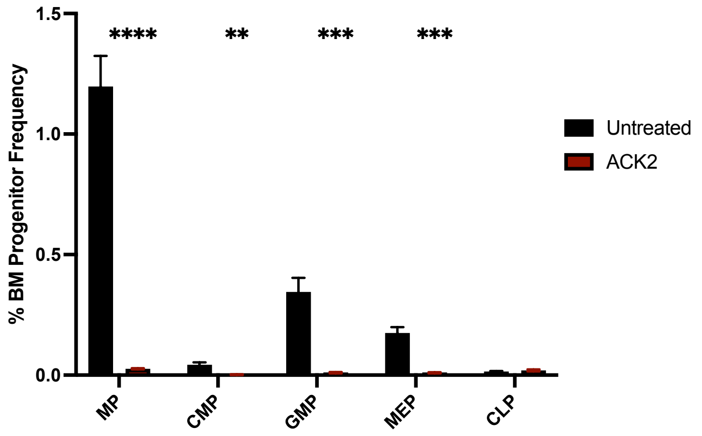

Supplemental Figure 2: Significant decrease of BM progenitors after αCD117 mAb ACK2 treatment in SCID settings. BM progenitors including myeloid progenitors (MyPro, MP), common myeloid progenitors (CMP), granulocyte-monocyte progenitors (GMP), megakaryocyte-erythrocyte progenitors (MEP), and common lymphoid progenitors (CLP) were measured 7 days after treatment with αCD117 mAb ACK2 in SCID mice. Statistics calculated using unpaired t-test compared with untreated controls (** $P < 0.01$; *** $P < 0.001$; **** $P < 0.0001$).

Supplemental Figure 3

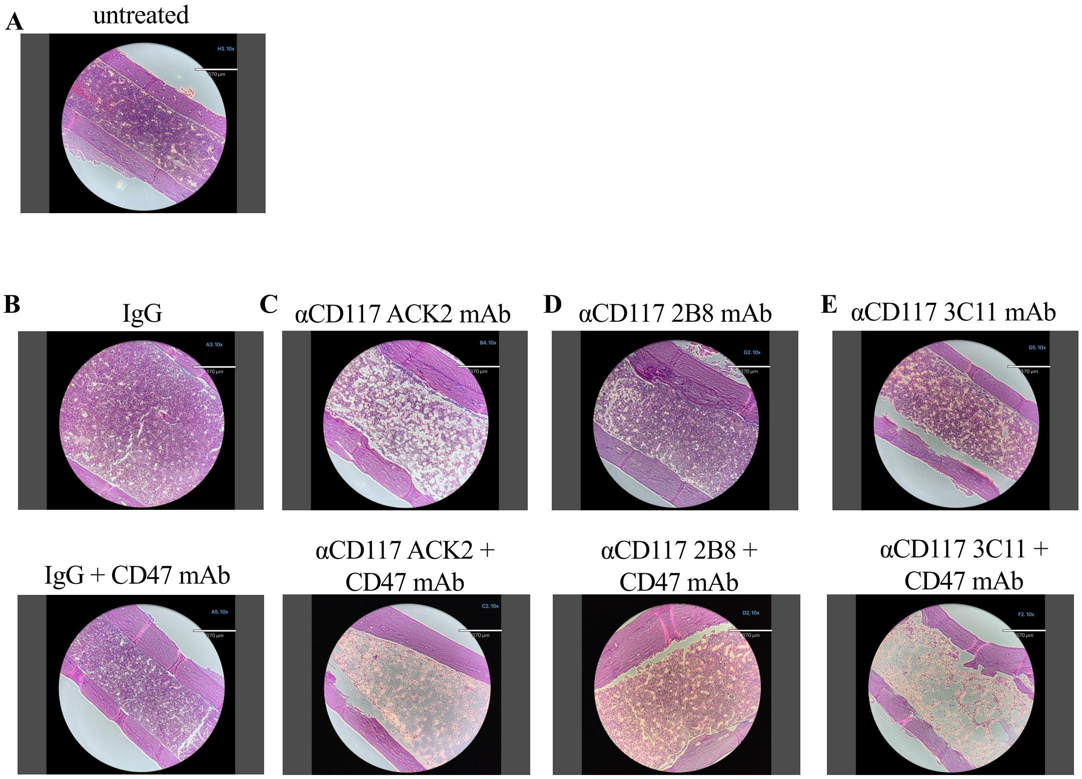

Supplemental Figure 3: Differential bone marrow effects observed after αCD117 mAbs augmented with αCD47 mAb treatment in WT mice. Representative bone marrow histology images obtained 7 days post treatment with 10X magnification for **(A)** untreated, **(B)** IgG and IgG+CD47 mAb, **(C)** αCD117 ACK2 mAb and αCD117 ACK2+CD47 mAb, **(D)** αCD117 2B8 mAb and αCD117 2B8+CD47 mAb, and **(E)** αCD117 3C11 mAb and αCD117 3C1+ CD47 mAb treatment groups. Profound BM aplasia was observed with both αCD117 ACK2+CD47 mAb and αCD117 3C11+CD47 mAb combination treatment.

Supplemental Figure 4

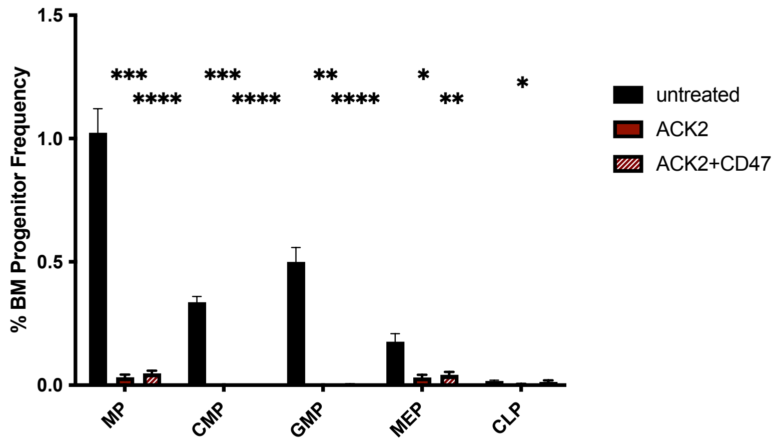

Supplemental Figure 4: Significant decrease in BM progenitors after αCD117 mAb ACK2 treatment with and without αCD47 mAb in WT settings. BM Progenitors of myeloid progenitors (MP), common myeloid progenitors (CMP), granulocyte-monocyte progenitors (GMP), megakaryocyte-erythrocyte progenitors (MEP), and common lymphoid progenitors (CLP) were measured 7 days after initiate conditioning with **(A)** αCD117 mAb ACK2 and αCD47 in WT mice (n=3-5). Statistics calculated using unpaired t-test compared with untreated controls (* $P < 0.05$; ** $P < 0.01$; *** $P < 0.001$; **** $P < 0.0001$).

Supplemental Figure 5

A

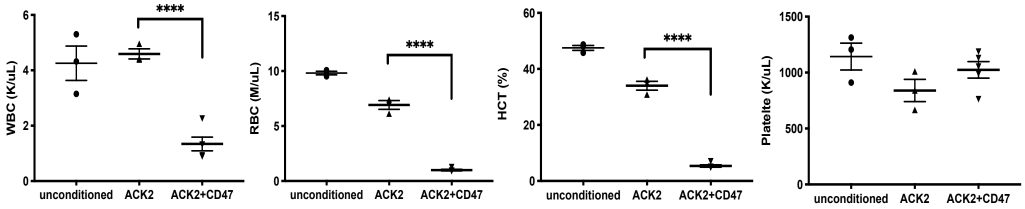

B

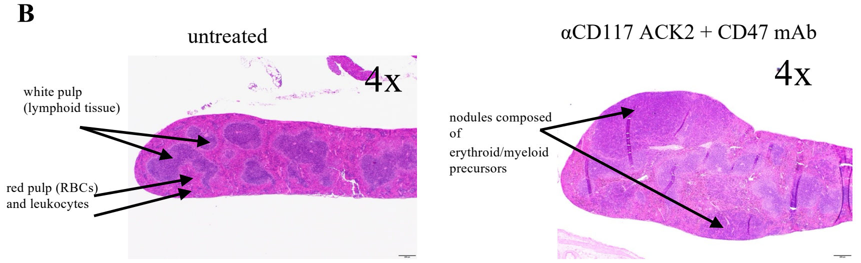

C

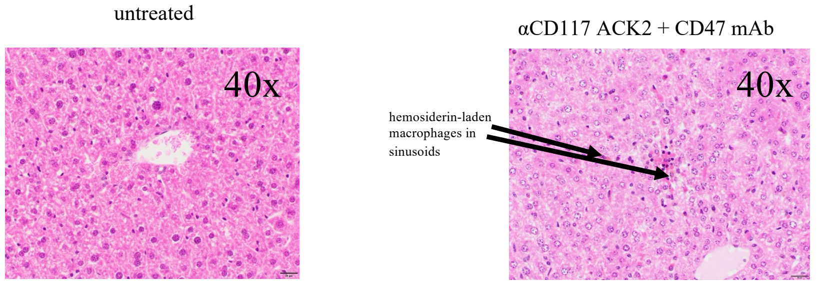

D

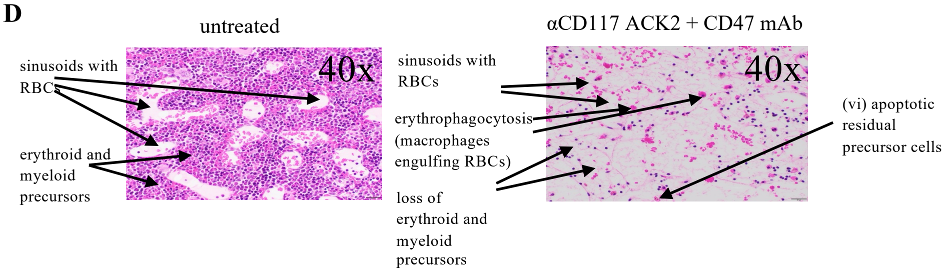

Supplemental Figure 5: Enhanced phagocytosis observed with addition of αCD47 mAb to αCD117 mAb ACK2 treatment in WT mice. WT mice were treated with αCD117 mAb ACK2 +/- αCD47 mAb. **(A)** Profound decrease in blood counts was observed in the αCD117 mAb ACK2 + CD47 mAb treated group at 7 days post treatment. Similarly, **(B)** Spleen at 4X magnification, **(C)** Liver at 40X magnification, and **(D)** BM at 40X magnification histology assessment via H+E staining showed apoptosis of various hematopoietic precursor cells 7 days post treatment.

Supplemental Figure 6

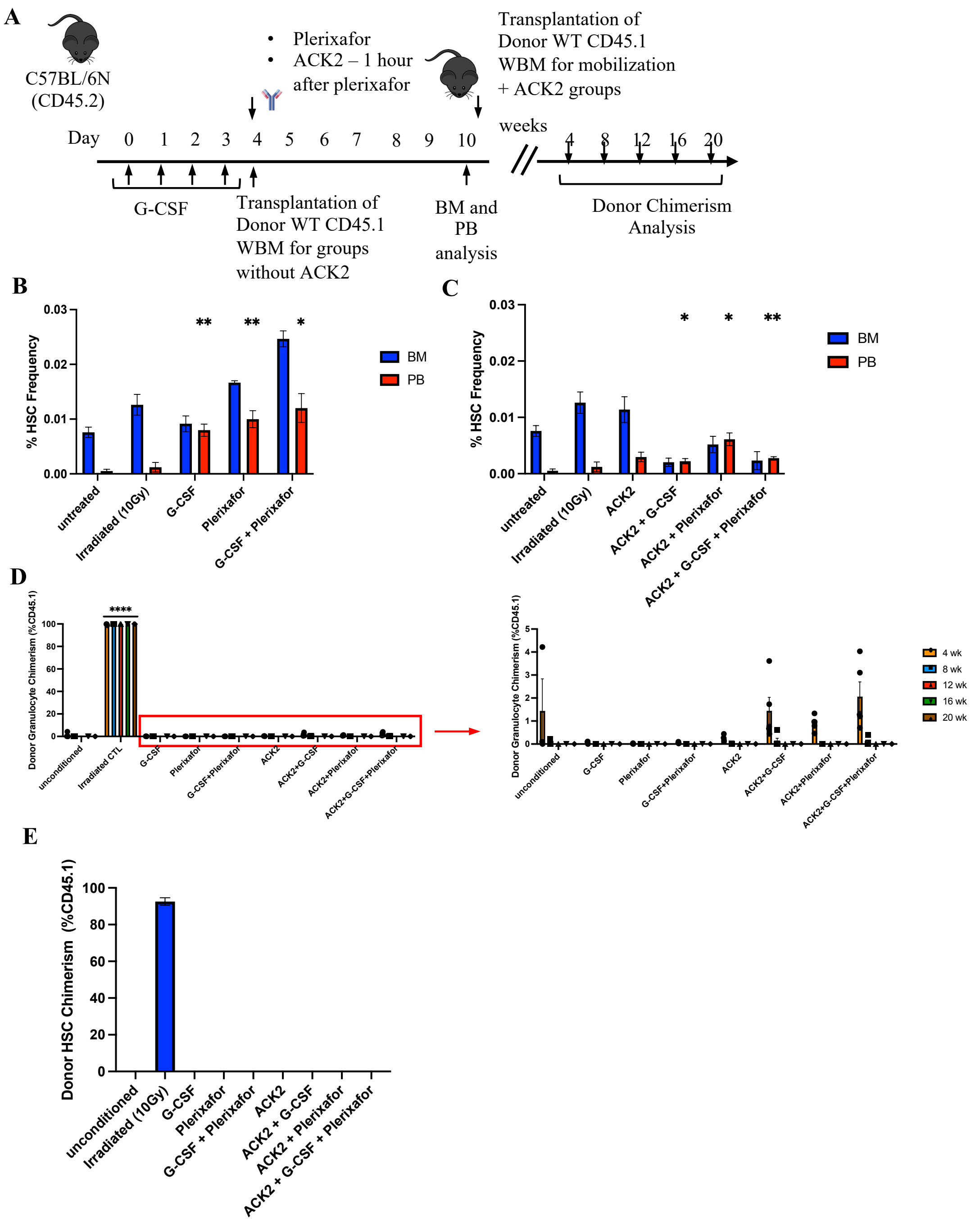

Supplemental Figure 6: Mobilization does not robustly enhance αCD117 mAb ACK2 BM HSC depletion or enable donor engraftment post HSCT in WT settings. (A) Experimental outline to illustrate treatment and assess efficacy of the combination of treatment with ACK2 and mobilization agents with G-CSF and plerixafor in WT mice. **(B)** BM HSC depletion was measured 4 days after treatment with mobilization agents G-CSF and plerixafor resulting in increased HSC frequency in BM and peripheral blood (PB). **(C)** Combination of mobilization agents and ACK2 resulted in decrease of HSC frequency in BM and increase of HSC in PB. **(D)** PB donor chimerism was measured up to 20 weeks post HSCT of conditioned animals with 10 x 10^6 WT CD45.1 donor WBM cells. The red box focus the analysis of lower engraftment level. **(E)** HSC donor chimerism was assessed 20 weeks post HSCT (n=5). Statistics calculated using unpaired t-test compared with unconditioned controls (* $P < 0.05$; ** $P < 0.01$; *** $P < 0.001$; **** $P < 0.0001$).

Supplemental Figure 7

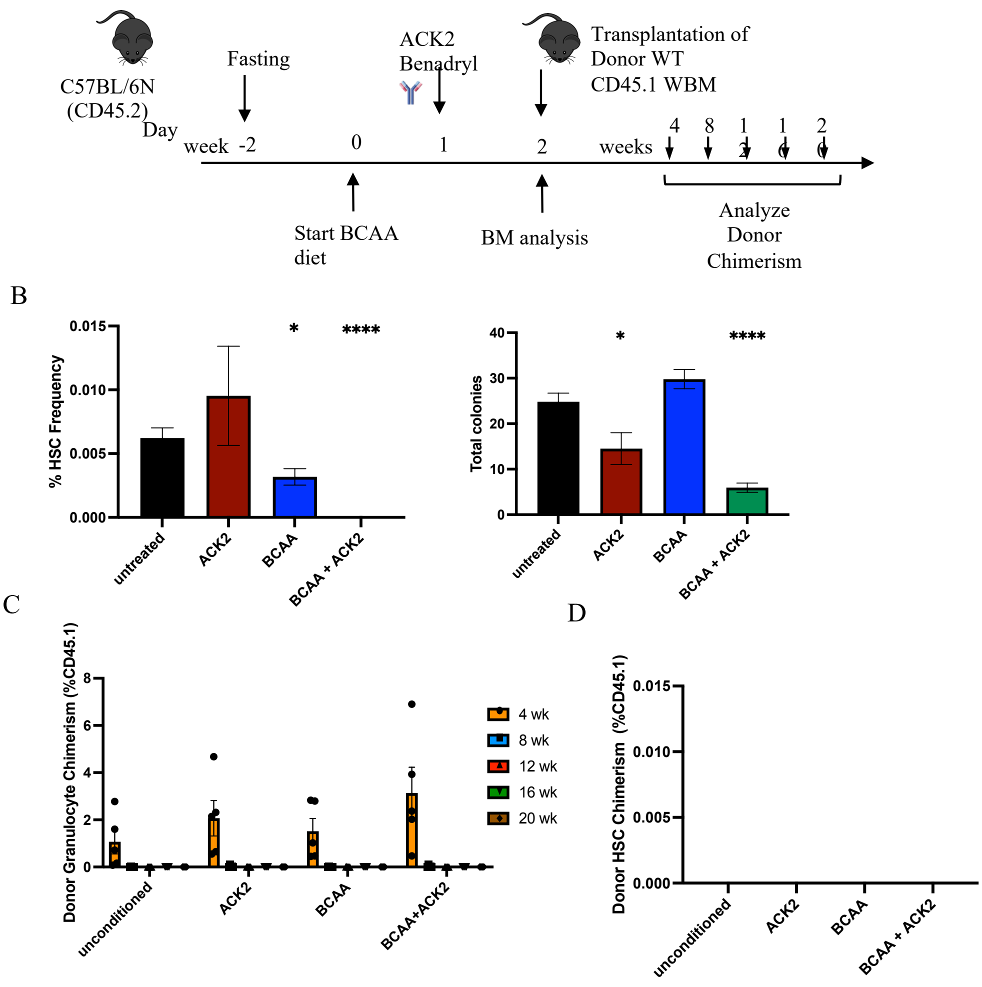

Supplemental Figure 7: Despite enhanced depletion of HSCs post αCD117 mAb ACK2 and BCAA diet, no enhancement in donor engraftment post HSCT in WT settings. (A) Experimental outline to illustrate and assess efficacy of the combination of ACK2 and BCAA diet in WT mice. **(B)** Assessment of BM HSC depletion was performed 5 days after treatment with ACK2 and 3.5 weeks after initiation of BCAA diet with profound observed depletion in animals treated with αCD117 ACK2 mAb augmented with BCAA diet. **(C)** Peripheral blood granulocyte and (**D)** BM HSC donor chimerism was measured up to 20 weeks post HSCT of conditioned animals with 10 x 10^6 WT CD45.1 donor WBM cells with no significant donor chimerism in any of the treated groups (n=5). Statistics calculated using unpaired t-test compared with unconditioned controls (* $P < 0.05$; ** $P < 0.01$; *** $P < 0.001$; **** $P < 0.0001$).

Supplemental Figure 8

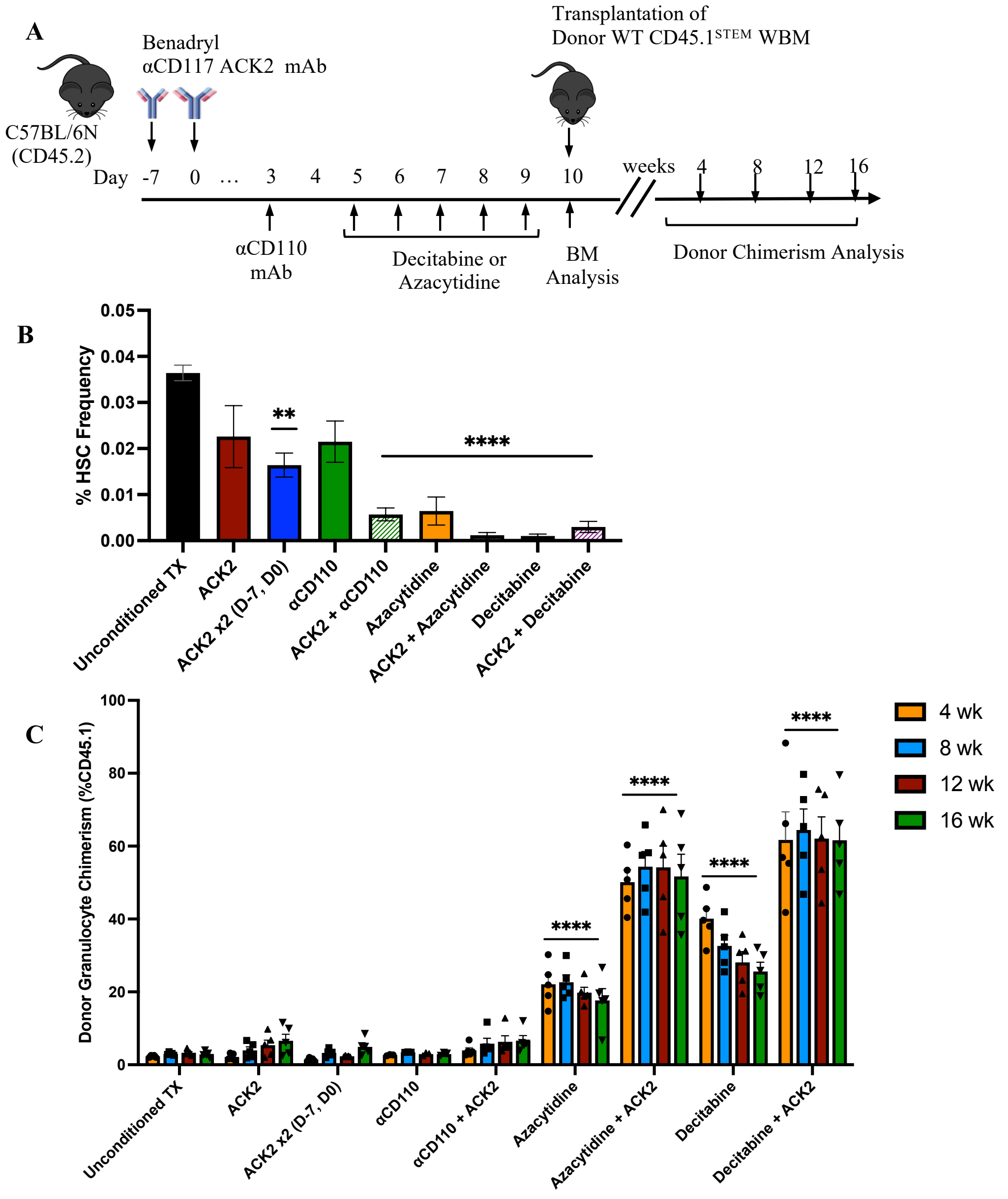

Supplemental Figure 8: HSC depletion with resulting robustly enhanced donor engraftment post HSCT in WT settings only observed with hypomethylating agents alone which was enhanced in combination with αCD117 mAb ACK2. (A) Experimental outline to assess mAb treatment, HSC depletion and HSCT transplantation efficacy with different combinations of αCD117 ACK2 mAb, αCD110 mAb, and/or DNA methyltransferase inhibitor (azacytidine or decitabine) treatment in WT mice. **(B)** BM HSC depletion was measured 10 days post treatment and phenotypic assessment was determined by flow cytometry ($Lin^{-}Sca\text{-}1^{+}CD117^{+}CD150^{+}CD48^{-}$). **(C)** PB donor chimerism was measured up to 12 weeks post HSCT of conditioned animals with 10 x 10^{6} WT CD45.1^{STEM} donor WBM cells. Robust donor engraftment was shown with azacytidine and decitabine alone and further improved in combination with αCD117 ACK2 (n=5). Statistics calculated using unpaired t-test compared with unconditioned controls (* $P < 0.05$; ** $P < 0.01$; *** $P < 0.001$; **** $P < 0.0001$).
